# Evaluating educational effectiveness of three-dimensional (3D)-printed training models and custom-made fibula model for osteotomy and flap inset training in head and neck reconstruction

**DOI:** 10.1186/s40902-025-00476-7

**Published:** 2025-08-20

**Authors:** Dharmik Gondalia, Mohit Parakh, Kunal Mokhale, Vineet Kumar, Ameya Bindu, Mayur Mantri, Saumya Mathews, Dushyant Jaiswal, Akshay Bhavke, Vinay Kant Shankhdhar

**Affiliations:** Department of Plastic and Reconstructive Surgery, Tata Memorial Hospital, Mumbai, India

**Keywords:** 3D-printed training model, Cost-effective, Osteotomy, Flap inset, Custom-made fibula model

## Abstract

**Background:**

Reconstructive surgery following head and neck cancer resection is inherently complex and technically demanding. Procedures such as osteotomy and flap inset involve a steep learning curve, yet opportunities for hands-on training are increasingly limited. Physical simulation using cost-effective, anatomically realistic models offers a promising solution. This study aimed to evaluate the educational value of 3D-printed training models and custom-made fibula models in enhancing surgical skills, supported by structured assessments and feedback.

**Methods:**

A hands-on workshop was conducted for 30 plastic surgery residents utilizing in-house 3D-printed models, created via fused deposition modeling (FDM), and acrylic-based fibula models. Participants performed simulated osteotomies and flap insets. Their performance was assessed using the 4-point Zwisch scale by two independent, blinded consultants. Additionally, a 6-item questionnaire was administered to capture self-reported improvements in anatomical understanding, surgical technique, and procedural planning. Pre- and post-training questionnaire scores were compared using Wilcoxon signed-rank test.

**Results:**

Post-training, the average questionnaire scores significantly improved from 12.03 ± 2.20 to 20.30 ± 1.56 (*p* < 0.01). The greatest improvement was noted in the participants’ comprehension of surgical planning. Zwisch scale evaluations demonstrated a clear progression toward greater technical independence. Participants also expressed high satisfaction with the anatomical realism, durability, and affordability of the training models.

**Conclusion:**

Low-cost 3D-printed training models and custom-made fibula models represent an effective and reproducible training tool for developing technical skills in head and neck reconstructive surgery. Their ease of fabrication, affordability, and anatomical accuracy make them particularly valuable in resource-limited settings. These models offer significant educational utility and warrant integration into structured surgical training curriculum.

## Background

Head and neck oncologic surgery often results in complex anatomical defects requiring intricate reconstruction to restore functions such as chewing, speech, and swallowing while also maintaining facial contour and structural balance [1]. These reconstructions, whether involving local, regional, or free tissue flaps, demand a deep understanding of anatomy and advanced technical expertise. Among the most technically challenging steps are bony osteotomies and precise flap inset, both of which critically influence functional and aesthetic outcomes. A properly executed flap inset reduces dead space, ensures oral competence, and minimizes complications such as orocutaneous fistula formation [2].

Traditionally, the progression to surgical competence follows an apprenticeship model, where trainees observe, assist, and gradually perform components of surgery under supervision. However, increased focus on patient safety, reduced operating room time, and inconsistent case exposure have significantly limited direct hands-on experience for residents [3]. To address these constraints, simulation-based surgical education has gained prominence. Borrowing from safety-driven fields like aviation, simulation allows learners to practice and make mistakes in a controlled, consequence-free environment — enhancing their competence and improving patient outcomes [4].

Emerging digital technologies, including augmented reality (AR), virtual reality (VR), and haptic-enabled platforms, offer immersive and visually rich environments for understanding spatial anatomy and procedural workflows. While promising, these tools are often limited by cost, accessibility, and inadequate tactile realism—especially in resource-limited settings [5, 6].

Despite digital advances, physical simulation models remain integral to surgical training. While animal cadavers have been historically used due to availability and affordability, ethical and institutional limitations now restrict their routine use [7]. Human cadaveric models provide superior anatomical fidelity but are associated with logistical, regulatory, and financial constraints. Commercial high-fidelity synthetic simulators offer reproducibility but often depend on proprietary materials and expensive infrastructure.

To address these barriers, additive manufacturing techniques such as fused deposition modeling (FDM) have taken attention as low-cost and adaptable solutions for creating custom anatomical models. FDM allows the transformation of computed tomography (CT) data into physical models using free, open-source design software, enabling institutions to develop patient-specific, reproducible training platforms [8].

Nonetheless, validated low-cost physical simulators for plastic and reconstructive surgery—especially those replicating both osteotomy and flap inset—are scarce. Furthermore, many existing models lack structured assessment frameworks to objectively track learner progress and surgical autonomy.

This study aims to address this gap by developing and evaluating a two-part simulation platform for reconstructive training in head and neck surgery: (1) A 3D-printed training model simulating head and neck oncologic defects for flap inset training and (2) a custom-made acrylic fibula model for practicing osteotomies.

## Materials and methods

This single-center prospective study was conducted at a tertiary care teaching hospital in India. A total of 30 plastic surgery residents were enrolled following an open call for voluntary participation. All participants provided written informed consent prior to the study. Since the research involved the use of anonymized CT imaging data and no identifiable patient information or clinical intervention, the ethical approval was waivered. A waiver was taken from Institutional Ethical Committee (IEC) for this study.

The primary objective of the study was to evaluate surgical skill development in two technically demanding tasks relevant to head and neck reconstruction: (1) Fibula osteotomy planning and execution and (2) soft tissue flap inset on 3D-printed models with bony and soft tissue defect. The study was simulation based and did not involve any live surgical procedures.

### 3D-printed training model: design and fabrication

CT scan images of a de-identified patient with a segmental mandibular defect were used to generate three-dimensional models. The images were converted from DICOM format to STL files using 3D Slicer, an open-source medical image segmentation software. Post-processing of the STL files was carried out using Blender to remove unwanted anatomical segments and Meshmixer to eliminate artifacts and refine the surface geometry. The finalized STL model was converted into a g-code format using Cura slicing software, enabling fabrication with a fused deposition modeling (FDM) 3D printer.

**Fig. 1:**
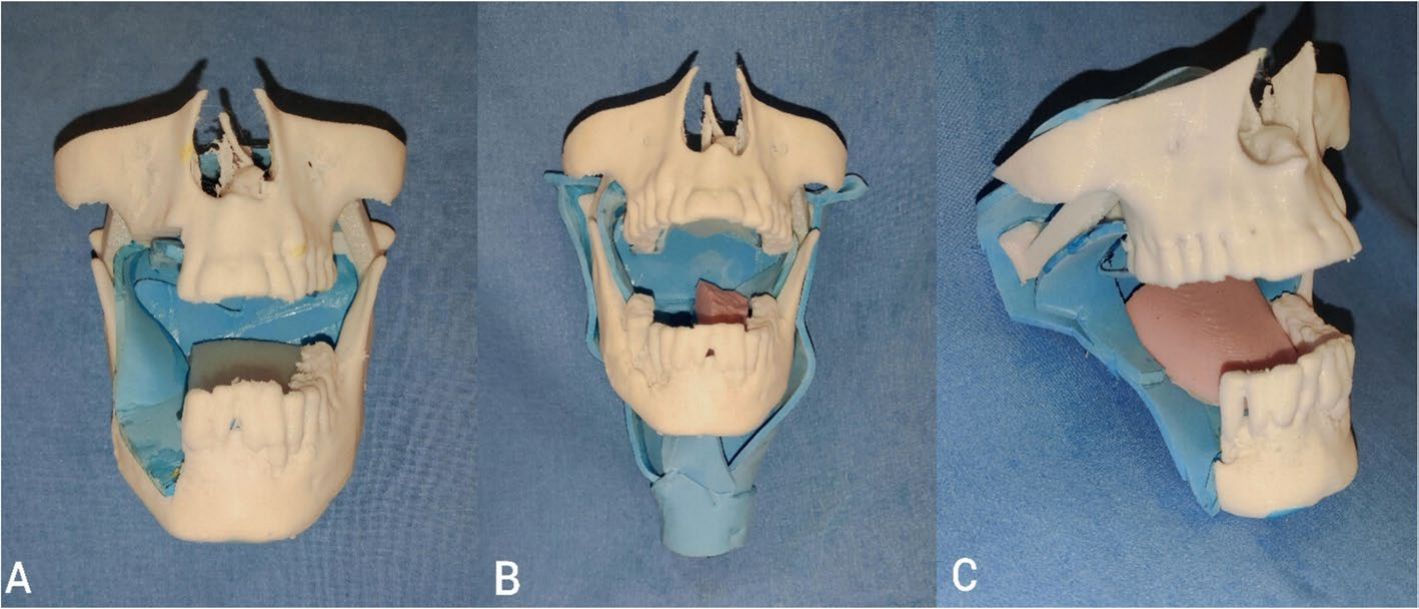
Composite image showing 3D-printed training models. **A** Marginal mandibulectomy with upper alveolectomy and resected buccal mucosa represented by blue foam. **B** Hemiglossectomy defect. The resected tongue is shown in brown, and surrounding buccal and pharyngeal mucosa are depicted using blue foam. **C** Posterior segmental mandibulectomy involving the posterior mandibular segment and buccal mucosa represented by blue foam

The in-house printer used had a build volume of 190 × 190 × 160 mm and nozzle diameter of 0.4 mm and supported PLA, PETG, and TPU filaments. Layer height ranged between 0.1 and 0.2 mm. Printing was carried out using PLA filament (1.75 mm) sourced from Solidspace Technology LLP, India. The printed mandible and maxilla were complemented by a simulated soft tissue component—namely, a tongue cast from platinum-grade silicone rubber using a 3D-printed mold. A mucosal lining was created using foam sheets, and all components were assembled with silicone-based adhesive. The silicone rubber was procured from Chemzest Techno products Pvt. Ltd. India. The cost per model was ₹1400 (~ US $16.39), with a breakdown as follows: PLA (150 g) ₹230, silicone (550 g) ₹900, silicone adhesive ₹100, and electricity used during 3D printing ₹170 (Fig. 1).

### Fibula model construction

**Fig. 2:**
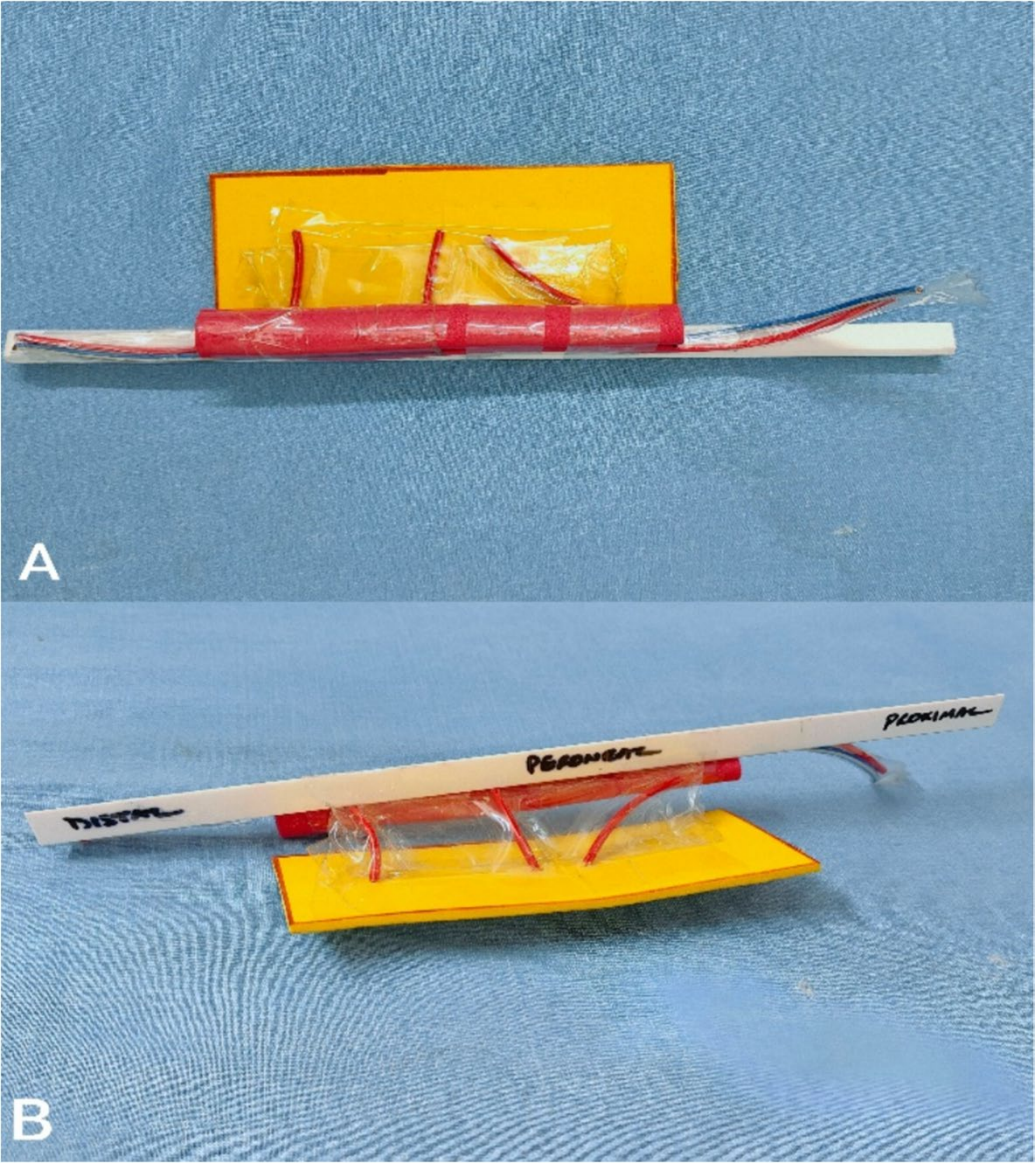
Composite image showing custom-made acrylic fibula model. **A** Acrylic white fibula segment with red foam representing the flexor hallucis longus (FHL), red copper wire mimicking vascular perforators, and yellow foam with transparent cellotape representing the skin paddle and septum. Blue and red copper wires represent the main pedicle. **B** Oblique view of the same model highlighting the anatomical relationship between fibula, muscle, perforator vessels, and septocutaneous paddle, designed to aid osteotomy and inset training

A 3D template of the fibula was derived from patient leg CT data and replicated using acrylic sheets. These sheets were manually cut into fibula shapes on a tilting table with a motorized cutting saw. The final construct had anatomical accuracy and similar density to bone, allowing for realistic osteotomy and plate fixation. The model incorporated several features as follows: Red foam to represent the flexor hallucis longus muscle, copper wire to mimic vascular perforators, yellow foam to simulate the skin paddle, and clear tape to replicate the interosseous septum. Each model cost ₹100 (~ US $1.17), with an additional ₹200 (~ US $2.34) spent on supporting stationery items (Fig. 2).

### Training setup

**Fig. 3:**
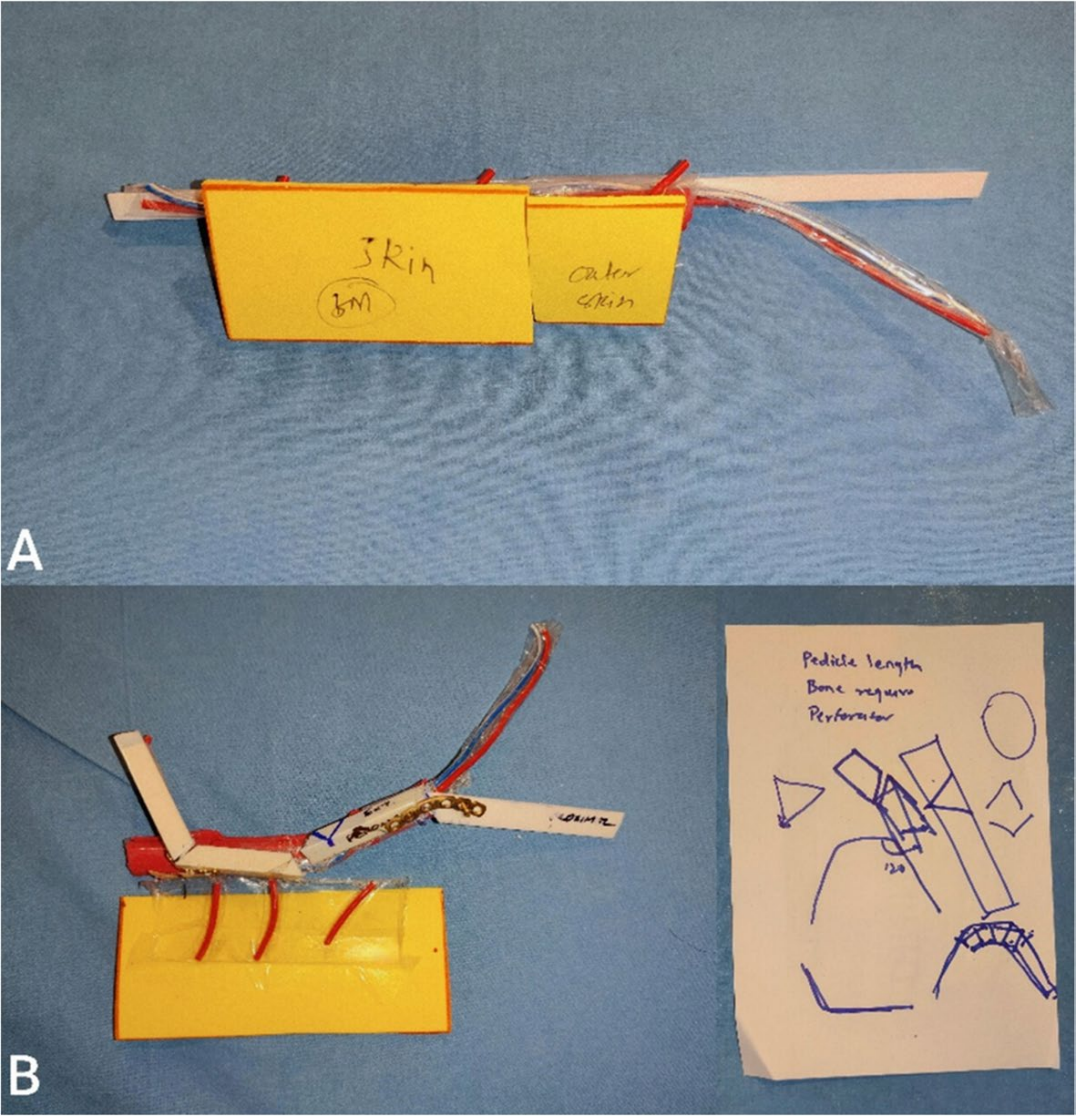
Composite image showing osteotomy planning and execution. **A** Custom-made acrylic fibula model before planning osteotomy. **B** Planning of osteotomy on the right side and fibula model after performing osteotomy on left

**Fig. 4:**
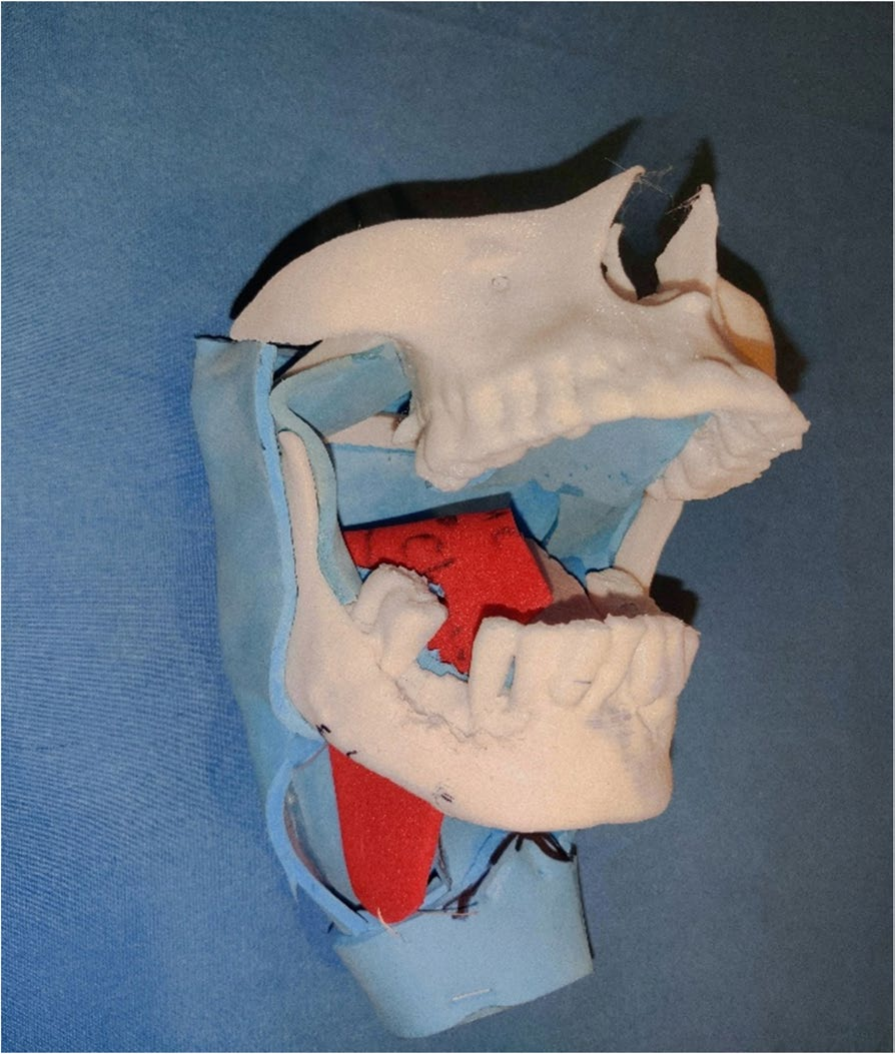
Image showing soft tissue inset of hemiglossectomy defect. Inset is represented by red foam

The training was delivered in two phases: an introductory lecture followed by two hands-on simulation modules. The first session focused on osteotomy planning and execution using the acrylic fibula constructs. The second involved flap inset onto the 3D-printed mandibular models, which included silicone-based soft tissues. Participants assembled their models using 1.5-mm titanium miniplates and screws and used standard surgical drills, burrs, and instruments under faculty supervision (Figs. 3 and 4).

### Assessment and evaluation

Participant performance was assessed using the Zwisch scale, a validated four-level scale developed to evaluate surgical autonomy in the context of competency-based education [9]. The scale includes Level 1 (“show and tell”), where the instructor performs and narrates the procedure; Level 2 (“active help”), where the trainee performs parts of the procedure with continuous instruction; Level 3 (“passive help”), where the trainee performs most steps independently with occasional input; and Level 4 (“supervision only”), where the trainee operates independently with the supervisor observing silently. Two blinded senior consultants independently evaluated each participant’s performance in three domains: fibula osteotomy execution, flap inset, and instrument handling. Discrepancies in scoring were resolved by consensus.

In addition to objective evaluation, participants completed a six-item questionnaire (Table 1) based on a 4-point Likert scale to assess self-perceived improvement in anatomical understanding, surgical technique, instrument use, and operative planning. This questionnaire format has been used in previous simulation-based education studies and includes statements ranging from “I was able to understand the contents of the lecture” to “I was able to understand the importance of surgical planning.”

**Table 1:**
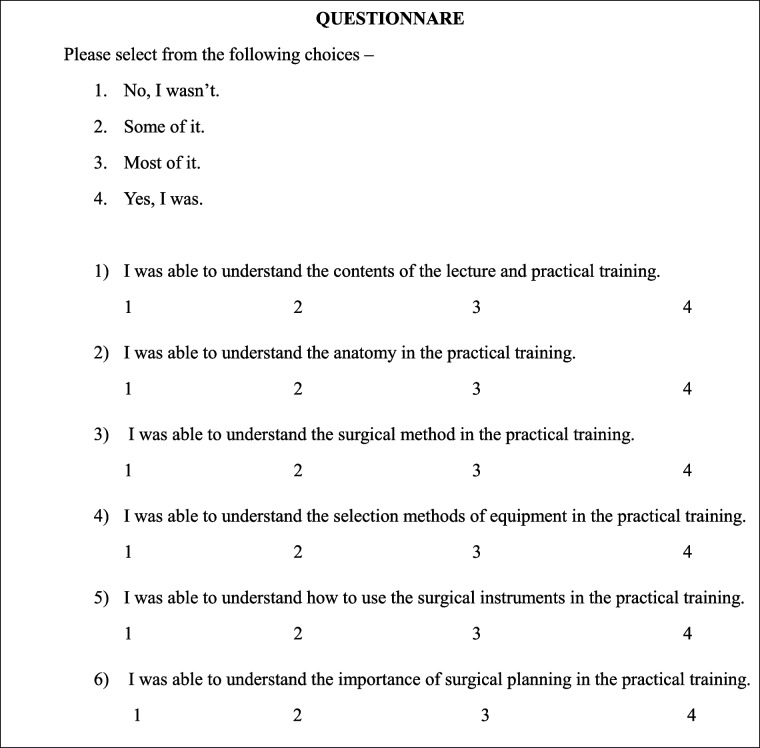
Six-item questionnaire

### Statistical analysis

All data were analyzed using IBM SPSS Statistics version 25.0. Pre- and post-training questionnaire scores were treated as paired ordinal data and compared using the Wilcoxon signed-rank test. Results were expressed as mean ± standard deviation, with *p*-values < 0.05 considered statistically significant.

## Results

A total of 30 plastic surgery residents participated in the simulation-based training and completed both pre- and post-intervention assessments. Analysis of the questionnaire responses revealed statistically significant improvement across all six assessed domains.

Pre-training mean scores for individual questions ranged from 1.87 to 2.50, while post-training mean scores ranged from 3.33 to 3.73. The most notable improvement was observed in the question related to understanding the importance of surgical planning (Q6), which increased from a mean score of 1.93 to 3.40. All six questions demonstrated statistically significant changes with *p*-values less than 0.01 when assessed using the Wilcoxon signed-rank test, confirming consistent improvement across all measured domains (Table 2).

**Table 2:** Pre- and post-assessment scores

Question	Pre-assessment Mean ± SD	Post-assessment Mean ± SD	*p*-value
Q1: I was able to understand the contents of the lecture and practical training	2.13 ± 0.57	3.27 ± 0.45	< 0.01
Q2: I was able to understand the anatomy in the practical training	2.10 ± 0.55	3.30 ± 0.47	< 0.01
Q3: I was able to understand the surgical method in the practical training	2.27 ± 0.53	3.47 ± 0.51	< 0.01
Q4: I was able to understand the selection methods of equipment in the practical training	1.87 ± 0.63	3.07 ± 0.58	< 0.01
Q5: I was able to understand how to use the surgical instruments	2.00 ± 0.61	3.17 ± 0.57	< 0.01
Q6: I was able to understand the importance of surgical planning	1.93 ± 0.60	3.40 ± 0.50	< 0.01

In terms of overall performance, the total mean questionnaire score increased from 11.62 ± 1.98 in the pre-training assessment to 20.11 ± 1.45 in the post-training assessment. The Wilcoxon signed-rank test confirmed that this improvement was statistically significant (*p* < 0.001) (Table 3). These findings suggest a substantial enhancement in self-reported understanding of surgical anatomy, technique, instrument handling, and planning following the simulation-based educational intervention.

**Table 3:** Pre- and post-training total score

Parameter	Pre-training score	Post-training score	Statistical test	*p*-value
Total mean score ± SD	11.62 ± 1.98	20.11 ± 1.45	Wilcoxon signed-rank test	< 0.001

## Discussion

In this study, simulation-based training using customized 3D-printed mandibular and fibula models resulted in significant improvements in self-reported understanding across all measured domains, with mean questionnaire scores rising from 11.62 ± 1.98 to 20.11 ± 1.45 (*p* < 0.001, Wilcoxon signed-rank test). These gains include knowledge of surgical anatomy, technique, instrument handling, and planning.

Our findings align well with recent evidence demonstrating educational and clinical benefits of 3D-printed models. Weber and colleagues evaluated the head-and-neck RACE model in microsurgical training and similarly reported that integrating anatomically realistic, spatially accurate models enhanced skill acquisition, learner engagement, and technical performance—particularly among residents who showed immediate improvements without initial skill decline [6, 10]. While their study used performance-based metrics like suturing time and spatial error, our study adds learner-perceived confidence and comprehension as valuable outcomes.

Clinically, studies on 3D-printed osteotomy guides have highlighted benefits in accuracy and operative efficiency. Jiang et al. showed shorter fold-down osteotomy planning times and improved fibula-to-defect ratios using guide-assisted techniques (1.32 vs. 1.62, *p* = 0.024) [11]. Another retrospective clinical cohort reported reduced overall operative and reconstruction times alongside enhanced postoperative alignment accuracy in the 3D-guided group versus controls [12]. Though our intervention was exclusively educational, the improved trainee comprehension of planning and technique implies greater readiness for real clinical procedures.

Meta-analyses on augmented and mixed reality in mandibular reconstruction also demonstrate that 3D-printed tools reduce angular osteotomy errors more reliably than AR/VR systems [13]. This further supports the suitability of physical 3D models in bridging simulation to improved surgical precision.

Notably, our most substantial gains were in self-reported surgical planning (mean score increased from 1.93 to 3.40). This aligns with clinical studies emphasizing preoperative planning as a driver of operative success—similar to reported efficiencies in ischemia time, operative speed, and precision when virtual planning is incorporated [14].

### Limitations

Study limitations include the single-group pre-/post-test design and reliance on subjective Likert-based metrics without objective task-level performance data. However, our results complement objective findings from performance-based and clinical studies, confirming the educational utility of 3D printing in reconstructive training. Future work should compare this platform against VR/AR alternatives and include objective metrics, such as plate fitting accuracy or procedure time, to better assess training transfer.

## Conclusion

In conclusion, our study demonstrates significant self-reported improvements in key reconstructive competencies following targeted simulation training using low-cost, anatomically realistic 3D-printed models and custom-made fibula models. This educational benefit complements existing literature on the clinical efficacy of similar technologies and supports the integration of 3D simulation tools into surgical curriculum.

## Data Availability

No datasets were generated or analysed during the current study.

## Abbreviations

3D: Three-dimensional
FDM: Fuse deposition modeling
CT: Computed tomography
AR: Augmented reality
VR: Virtual reality
IEC: Institutional Ethical Committee
